# Male Obesity and Cardiometabolic Risk: Inflammatory Mechanisms and Clinical Implications

**DOI:** 10.3390/biomedicines14071414

**Published:** 2026-06-23

**Authors:** Rodolfo de Oliveira Medeiros, Cristiano Machado Galhardi, Carlos Horacio Vargas Urzagaste, Camila Menon Oliveros, Gustavo Silveira Pires, Vinícius Willian Calderon da Silva, Felipe Quieregati de Novais, Isabela Gazola Suzuki, Hugo Calesso dos Reis, José Antonio Pizzolato Neto, Felipe Ravazzi Guzzo, Marcus Vinicius da Silva Zanelato, Rafael Ignácio dos Santos, Pedro Henrique Lima Domingues, Bruna Gonçalves Manzoni, Melissa Antunes, Teófilo Augusto Araújo Tiradentes, Victor Cáppia, Thiago Luengo Tavares, Altair Martins Barasuol

**Affiliations:** 1 Department of Medicine, University of Marília (UNIMAR), Marília 17525902, SP, Brazil; drgalhardi@outlook.com.br (C.M.G.); carloshoraciovargasurzagaste@gmail.com (C.H.V.U.); cmo1406@gmail.com (C.M.O.); gustavospires207@gmail.com (G.S.P.); vinicius.will42@gmail.com (V.W.C.d.S.); felipe.quieregati@gmail.com (F.Q.d.N.); isabelasuzuki463@gmail.com (I.G.S.); mrreisbr@gmail.com (H.C.d.R.); netopizzolato@gmail.com (J.A.P.N.); feliperavazzi2020@gmail.com (F.R.G.); marcuszanelato76@gmail.com (M.V.d.S.Z.); rafa_ynacio@hotmail.com (R.I.d.S.); pedrin_lima@hotmail.com (P.H.L.D.); bruninha-gm@hotmail.com (B.G.M.); 2 Barretos School of Health Sciences (FACISB), Barretos 3505708, SP, Brazil; melissa.antunes83@gmail.com; 3 Irmandade da Misericórdia da Santa Casa de Marília, Marília 3529005, SP, Brazil; teofilo.augusto@unesp.br (T.A.A.T.); victor_cappia@hotmail.com (V.C.); thiago.luengo.tavares@gmail.com (T.L.T.); altair92medufms@gmail.com (A.M.B.)

**Keywords:** male obesity, cardiometabolic risk, inflammation, visceral adiposity

## Abstract

Obesity is a major global health challenge strongly associated with increased cardiometabolic morbidity and mortality. In men, obesity is characterized by a predominance of visceral adiposity, which is metabolically active and closely linked to systemic inflammation, hormonal dysregulation, and adverse cardiovascular outcomes. Despite its clinical relevance, male obesity remains underrecognized as a distinct pathophysiological condition. This study aimed to analyze the inflammatory mechanisms underlying male obesity and their relationship with cardiometabolic risk. A structured narrative review was conducted based on a PICo-guided research question, with literature searches performed in PubMed/MEDLINE, Scopus, Web of Science, Embase, and ScienceDirect, covering publications from 2015 to 2026. Studies focusing on male obesity, inflammatory pathways, and cardiometabolic outcomes were included. Evidence indicates that visceral adipose tissue acts as an active endocrine organ, releasing pro-inflammatory cytokines such as TNF-α and IL-6, contributing to chronic low-grade inflammation. This inflammatory state is associated with insulin resistance (IR), endothelial dysfunction, and oxidative stress, mediated by intracellular pathways including NF-κB and JNK. Additionally, adipokine imbalance, characterized by reduced adiponectin and increased leptin levels, further exacerbates metabolic and vascular impairment. Hormonal alterations, particularly reduced testosterone levels, play a key role in amplifying visceral fat accumulation and inflammation, creating a bidirectional relationship between hypogonadism and metabolic dysfunction. Clinically, these mechanisms highlight the importance of integrating inflammatory biomarkers, body composition assessment, and hormonal evaluation into the management of male obesity. Emerging therapies, including GLP-1 receptor agonists and immunometabolic interventions, offer promising strategies for reducing cardiometabolic risk. In conclusion, male obesity represents a complex, inflammation-driven condition requiring a comprehensive and mechanism-based approach to improve clinical outcomes and guide future therapeutic developments.

## 1. Introduction

Obesity represents one of the major contemporary public health challenges, with a growing impact on global morbidity and mortality, particularly due to its strong association with cardiovascular and metabolic diseases. Although obesity has been extensively investigated from a general perspective, emerging evidence suggests that it exhibits distinct pathophysiological characteristics between sexes, while obesity in men often remains underdiagnosed and underrecognized in prevention and clinical management strategies [1,2].

In men, visceral fat accumulation predominates, exhibiting high metabolic activity and a strong association with adverse cardiometabolic outcomes. This pattern of fat distribution promotes a systemic pro-inflammatory environment, endothelial dysfunction, and hormonal alterations, including reduced testosterone levels. These factors interact in a complex and interconnected manner, contributing to the development of conditions such as IR, type 2 diabetes mellitus, hypertension, and atherosclerosis [3,4,5].

Age-related declines in testosterone levels may further contribute to visceral fat accumulation and metabolic dysfunction in men. Expanded adipose tissue exhibits increased aromatase activity, promoting the peripheral conversion of testosterone into estrogen, which may aggravate hormonal imbalance and favor the progression of adiposity and chronic inflammation. This bidirectional interaction between obesity and hypogonadism reinforces the complex endocrine–metabolic mechanisms underlying male cardiometabolic risk [4,5].

From a biological perspective, male obesity should be understood as a chronic low-grade inflammatory condition characterized by the continuous secretion of adipokines and pro-inflammatory cytokines capable of modulating critical cellular and molecular pathways. This persistent inflammatory state establishes a dynamic interface among metabolism, immune function, and vascular homeostasis, playing a central role in the development of cardiometabolic risk [6,7].

In this context, a comprehensive understanding of the inflammatory mechanisms associated with male obesity and their clinical implications is essential. Therefore, the present study aims to analyze the main inflammatory mechanisms involved in male obesity and their relationship with cardiometabolic risk, highlighting clinical implications and emerging therapeutic perspectives.

## 2. Methods

This study is characterized as a structured narrative review, conducted with a rigorous and transparent methodological approach aimed at ensuring consistency, analytical depth, and reproducibility in the synthesis of scientific evidence. Although not designed as a systematic review, the methodological framework was informed by established principles commonly adopted in integrative and evidence-based reviews, allowing for a comprehensive and critically oriented analysis of the available literature [8].

The guiding research question was developed based on an adaptation of the PICo strategy and was defined as follows: “What are the main inflammatory mechanisms associated with male obesity, and how do these mechanisms influence cardiometabolic risk?” Within this framework, the population (P) consisted of men with obesity, the phenomenon of interest (I) referred to inflammatory mechanisms, and the context (Co) corresponded to the development of cardiometabolic outcomes. The use of a structured research question was intended to enhance analytical clarity, improve the organization of evidence, and support a focused and coherent synthesis process [9,10].

The literature search was conducted across major biomedical databases, including PubMed/MEDLINE, Scopus, and Web of Science, selected for their broad coverage and high-quality indexing of peer-reviewed studies. To increase search sensitivity and ensure comprehensive coverage of the topic, additional databases such as ScienceDirect and Embase were also consulted. This multi-database approach was designed to minimize selection bias and maximize the inclusion of relevant scientific evidence.

Controlled descriptors and keywords were systematically combined using Boolean operators to optimize the search strategy. The primary terms included “male obesity”, “visceral adiposity”, “inflammation”, “cytokines”, “cardiometabolic risk”, “insulin resistance”, and “endothelial dysfunction”. The search was limited to studies published between 2015 and 2026, prioritizing recent and high-impact publications, including original research articles, narrative and systematic reviews, and relevant clinical guidelines.

The inclusion criteria comprised studies specifically addressing obesity in men or presenting sex-stratified analyses, with an emphasis on inflammatory mechanisms and their association with cardiometabolic outcomes. Exclusion criteria included studies focused exclusively on female populations, investigations with a purely behavioral approach without mechanistic or biological analysis, and articles lacking full-text availability. These criteria were defined to ensure the relevance and scientific robustness of the selected evidence.

The initial search identified 742 records across the selected databases. After duplicate removal, 528 records remained for title and abstract screening. Of these, 312 records were excluded for not meeting the predefined eligibility criteria. A total of 216 full-text articles were assessed for eligibility, and 168 were excluded due to population mismatch, lack of mechanistic focus, predominantly behavioral approaches, or lack of full-text availability. Finally, 48 studies were included in the qualitative synthesis. Although this review was not designed as a systematic review, the selection process was organized in a PRISMA-inspired flow diagram to improve reproducibility and methodological clarity.

**Figure 1:**
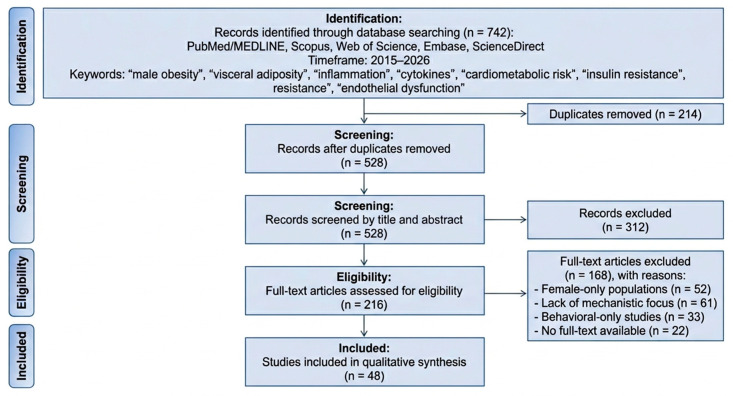
Study Selection Process Diagram. Source: Developed by the authors based on the study selection strategy.

The study selection process was conducted through a structured and sequential approach, involving the evaluation of titles, abstracts, and full texts. This process was guided by predefined criteria to ensure consistency and methodological coherence in the identification of relevant studies. Although the review does not follow a formal systematic design, the selection pathway was organized to enhance clarity and traceability in the inclusion of evidence, as illustrated in Figure 1.

Data analysis was performed using a descriptive and interpretative approach, with thematic organization based on key mechanistic and clinical domains. This strategy enabled the integration of findings from different types of studies, supporting a comprehensive understanding of the immunometabolic interactions underlying male obesity and their implications for cardiometabolic risk.

The main studies analyzed throughout this narrative review are summarized in Supplementary Table S1.

## 3. Pathophysiological Mechanisms

### 3.1. Visceral Adiposity and Chronic Low-Grade Inflammation

Male obesity is predominantly characterized by the accumulation of visceral adipose tissue, which exhibits high metabolic and secretory activity and plays a central role in systemic metabolic dysregulation. Unlike subcutaneous fat, visceral adipose tissue acts as an active endocrine organ, releasing a broad range of adipokines, chemokines, and pro-inflammatory cytokines, including TNF-α, IL-6, and C-reactive protein (CRP), all of which contribute to the development and persistence of a chronic low-grade systemic inflammatory state [3,6,7]. This persistent inflammatory environment reflects a complex interplay between metabolic overload and immune system activation, positioning visceral adiposity as a major driver of immunometabolic dysfunction [11,12].

**Figure 2:**
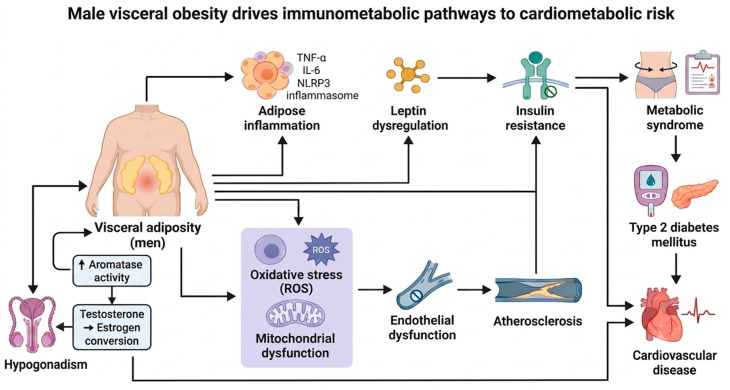
Immunometabolic mechanisms linking male obesity to cardiometabolic risk. Visceral adiposity promotes chronic low-grade inflammation, adipokine dysregulation, inflammasome activation, oxidative stress, mitochondrial dysfunction, endothelial injury, IR, and hypogonadism, contributing to metabolic syndrome, type 2 diabetes mellitus, atherosclerosis, and cardiovascular disease. Created with BioRender.com. Medeiros, R. (2026). https://app.biorender.com/illustrations/69fd55c16b1c31fc3998061d (accessed on 29 May 2026).

The complex interaction among visceral adiposity, chronic inflammation, oxidative stress, hormonal dysregulation, and cardiometabolic complications in male obesity is summarized in Figure 2.

Recent evidence has further demonstrated that the inflammatory activity of visceral adipose tissue is closely associated with activation of the NLRP3 inflammasome, an intracellular multiprotein complex involved in innate immune signaling and chronic metabolic inflammation. Activation of this pathway promotes the maturation and release of pro-inflammatory cytokines, particularly IL-1β and IL-18, which contribute to systemic IR, endothelial dysfunction, and metabolic deterioration in obesity [13,14]. In men, the predominance of visceral adiposity appears to intensify this inflammatory activation, further reinforcing the association between abdominal obesity and increased cardiometabolic vulnerability.

Additionally, obesity-induced adipose tissue remodeling is accompanied by profound alterations in immune cell composition. Besides macrophage polarization toward the pro-inflammatory M1 phenotype, recent studies have identified significant participation of neutrophils, dendritic cells, mast cells, and T lymphocyte subpopulations in the perpetuation of chronic adipose tissue inflammation [15]. The interaction between innate and adaptive immune responses contributes to a sustained inflammatory microenvironment that amplifies metabolic dysfunction and promotes progressive vascular injury.

### 3.2. Oxidative Stress, Mitochondrial Dysfunction, and Ectopic Fat Accumulation

Another relevant mechanism involves mitochondrial dysfunction and impaired cellular energy homeostasis. In obesity, excessive nutrient availability and lipotoxicity lead to mitochondrial stress, reduced oxidative phosphorylation efficiency, and excessive production of reactive oxygen species (ROS). These alterations contribute not only to oxidative damage but also to further activation of inflammatory signaling pathways, including NF-κB and inflammasome-mediated responses [16]. This interaction establishes a vicious cycle between oxidative stress and inflammation, which plays a central role in the progression of obesity-related cardiometabolic complications.

Furthermore, growing evidence suggests that ectopic lipid accumulation in organs such as the liver, skeletal muscle, and myocardium significantly contributes to systemic metabolic dysfunction. Lipid overflow resulting from dysfunctional adipose tissue promotes local inflammatory activation and tissue-specific IR, thereby aggravating cardiometabolic risk and increasing susceptibility to conditions such as nonalcoholic fatty liver disease, cardiac remodeling, and metabolic syndrome [17]. These findings reinforce the concept that obesity should be understood as a multisystem immunometabolic disorder rather than merely a condition characterized by excess body weight.

### 3.3. Insulin Resistance, Endothelial Dysfunction, and Adipokine Imbalance

Hypertrophied adipocytes further contribute to adipose tissue dysfunction through local hypoxia, mechanical stress, and sustained inflammatory activation. These alterations intensify immune cell recruitment and perpetuate chronic low-grade inflammation, reinforcing adipose tissue as an active immunometabolic organ [6,18].

This inflammatory environment promotes alterations in insulin signaling, primarily through the activation of intracellular pathways such as NF-κB and JNK, which interfere with insulin receptor substrate phosphorylation and impair downstream signaling cascades. As a consequence, IR develops, leading to reduced glucose uptake, hyperglycemia, and compensatory hyperinsulinemia. These metabolic disturbances extend beyond glucose metabolism, contributing to lipid dysregulation and ectopic fat deposition, further aggravating systemic metabolic imbalance [3,7,11,19].

In parallel, the resulting metabolic dysfunction directly affects endothelial function by reducing nitric oxide bioavailability, increasing oxidative stress, and promoting vascular inflammation. These alterations impair vasodilation, increase vascular stiffness, and facilitate the adhesion of inflammatory cells to the endothelium, thereby accelerating the progression of atherosclerosis and increasing cardiovascular risk [3,7,19]. The interplay between metabolic and vascular dysfunction further highlights the systemic nature of obesity-related inflammation.

Additionally, an imbalance in adipokine secretion is observed, characterized by reduced adiponectin levels, an adipokine with anti-inflammatory, insulin-sensitizing, and cardioprotective properties, and increased leptin levels, which, when chronically elevated, are associated with leptin resistance, persistent inflammatory signaling, endothelial dysfunction, and sympathetic nervous system activation. In addition, elevated resistin levels may further contribute to IR and vascular inflammation, reinforcing metabolic and cardiovascular impairment. This dysregulated adipokine profile contributes to the persistence of the inflammatory state and further amplifies cardiometabolic risk through both metabolic and vascular pathways [3,7,12].

### 3.4. Hormonal Dysregulation and Male Hypogonadism

Another relevant aspect involves the interaction between obesity and the male hormonal axis. Reduced testosterone levels, commonly observed in men with obesity, are associated with increased visceral adiposity and an intensified inflammatory response. This establishes a bidirectional relationship between functional hypogonadism and metabolic inflammation, in which decreased androgen levels favor adipose tissue expansion, while adiposity-related inflammation further suppresses gonadal function. This endocrine–metabolic interplay represents a critical mechanism underlying the progression of male obesity and its associated complications [4,5,20].

Furthermore, oxidative stress emerges as a key component of this pathophysiological process. The excessive production of ROS, largely driven by mitochondrial dysfunction and chronic inflammation, contributes to cellular damage, lipid peroxidation, and further activation of inflammatory pathways. This creates a feedback loop in which oxidative stress and inflammation mutually reinforce each other, exacerbating endothelial dysfunction and metabolic impairment [6,18].

### 3.5. Integrated Immunometabolic Interactions and Cardiometabolic Consequences

Collectively, these interconnected mechanisms illustrate that male obesity is not merely a condition of excess adiposity, but rather a complex immunometabolic disorder characterized by the integration of inflammatory, hormonal, and metabolic pathways. Based on the analysis of the selected studies, it was possible to systematize the main inflammatory mechanisms, biological mediators, and their clinical repercussions, providing an integrated view of the interactions between male obesity and cardiometabolic risk.

Taken together, these mechanisms demonstrate that male obesity represents a multifactorial immunometabolic condition characterized by the convergence of endocrine, inflammatory, vascular, and metabolic dysregulation. The predominance of visceral adiposity in men may intensify these interactions through enhanced inflammatory signaling and increased ectopic fat deposition, helping to explain sex-related differences in cardiometabolic vulnerability. The main inflammatory and immunometabolic mechanisms linking male obesity to cardiometabolic risk are summarized in Table 1.

## 4. Clinical Implications

The pathophysiological alterations associated with male obesity have direct and clinically relevant implications, particularly regarding risk stratification, early diagnosis, and the comprehensive management of cardiometabolic diseases (CMDs). Recognizing the central role of chronic low-grade inflammation in obesity enables a more integrated clinical approach that extends beyond traditional assessments based solely on anthropometric indices such as Body Mass Index (BMI) [2,24].

The predominance of visceral adiposity in men reinforces the need for complementary diagnostic measures, including waist circumference, waist-to-hip ratio, and imaging-based body composition assessments, to improve the accuracy of cardiometabolic risk evaluation. These tools allow for a more precise identification of individuals at higher risk, particularly those with metabolically active adipose tissue, which is strongly associated with adverse outcomes [1,2,24].

In addition to anthropometric measures, the incorporation of inflammatory biomarkers into clinical practice represents a promising strategy for refining risk stratification. Markers such as CRP, IL-6, and other cytokines may provide valuable insights into the inflammatory status of patients and help identify individuals at increased risk for cardiovascular events. Although not yet universally implemented, the use of such biomarkers may contribute to a more personalized and mechanism-based approach to patient management [1,2,24].

The association between obesity and functional hypogonadism also has significant clinical implications. Reduced testosterone levels are consistently linked to an unfavorable metabolic profile, increased IR, and elevated cardiovascular risk. This relationship highlights the importance of considering hormonal evaluation as part of the clinical assessment in men with obesity, particularly in cases of severe metabolic dysfunction or refractory disease. However, therapeutic decisions should remain individualized, taking into account potential risks and benefits, as well as the presence of comorbidities [5,20,25].

From a therapeutic perspective, interventions aimed at reducing inflammation and excess adiposity, including lifestyle modifications, nutritional strategies, and physical activity, remain the cornerstone of clinical management. These interventions not only promote weight loss but also improve metabolic parameters and reduce systemic inflammation. Importantly, their benefits extend beyond weight reduction, contributing to improved endothelial function, insulin sensitivity, and overall cardiovascular health [2,20].

In this context, a shift toward a more integrative clinical model is necessary, one that recognizes obesity as a systemic inflammatory disease and incorporates metabolic, hormonal, and inflammatory dimensions into patient care. Such an approach may enhance clinical outcomes and support more effective long-term management strategies.

An additional aspect that deserves attention involves the heterogeneity of obesity phenotypes and their distinct cardiometabolic implications. Not all individuals with obesity exhibit the same inflammatory and metabolic profiles, and increasing evidence supports the existence of metabolically unhealthy obesity phenotypes characterized by greater visceral adiposity, systemic inflammation, and endothelial dysfunction [17,22]. In men, this heterogeneity appears to be particularly influenced by hormonal status, fat distribution patterns, and inflammatory burden, reinforcing the importance of individualized clinical assessment.

Moreover, the integration of biomarkers into routine clinical evaluation may contribute to the early identification of patients at elevated cardiometabolic risk, even before the onset of overt metabolic disease. The incorporation of inflammatory and hormonal parameters into obesity management may allow a more precise characterization of disease severity and facilitate personalized therapeutic interventions [23]. In this context, precision medicine approaches are increasingly being explored as strategies to optimize cardiometabolic prevention and improve long-term outcomes in patients with obesity.

Nevertheless, despite the growing relevance of inflammatory biomarkers in obesity-related cardiometabolic risk stratification, important limitations remain. Biomarkers such as CRP, IL-6, and TNF-α may exhibit considerable interindividual variability and are influenced by multiple metabolic, genetic, hormonal, and environmental factors, which may limit their specificity and clinical reproducibility. In addition, not all individuals with obesity present the same inflammatory or metabolic profile, reinforcing the heterogeneity of obesity phenotypes and the complexity of translating biomarker-based approaches into routine clinical practice. These aspects highlight the need for cautious interpretation of inflammatory markers and for more standardized approaches in obesity-related risk assessment [11,12,17,22,23].

The psychosocial burden associated with male obesity should also be considered within a comprehensive clinical framework. Obesity in men is frequently associated with reduced quality of life, depressive symptoms, social stigma, and decreased adherence to long-term lifestyle interventions [26]. These psychosocial factors may indirectly contribute to worsening metabolic control and reduced therapeutic effectiveness, highlighting the importance of multidisciplinary and patient-centered approaches in obesity management.

The main clinical biomarkers and parameters with potential relevance for cardiometabolic risk stratification in male obesity are summarized in Table 2.

## 5. Emerging Therapies and Future Directions

Recent advances in the understanding of inflammatory mechanisms associated with obesity have driven the development of novel therapeutic approaches that target both metabolic and immune pathways. Among these, glucagon-like peptide-1 (GLP-1) receptor agonists have emerged as one of the most promising pharmacological strategies, demonstrating consistent efficacy in promoting weight reduction, improving glycemic control, and reducing cardiovascular risk in individuals with overweight or obesity [27,28].

Beyond their metabolic effects, GLP-1 receptor agonists have also been shown to exert anti-inflammatory actions, including the modulation of cytokine production and improvement of endothelial function. These properties highlight their potential role as immunometabolic agents, capable of addressing both the metabolic and inflammatory components of obesity [27,28].

In addition to GLP-1-based therapies, other pharmacological approaches targeting IR and cardiovascular risk, such as sodium–glucose cotransporter-2 (SGLT2) inhibitors, have gained increasing attention. Although primarily developed for glycemic control, these agents have demonstrated significant cardiovascular and renal benefits, suggesting a broader role in the management of CMDs [27,28,29].

The modulation of adipose tissue inflammation through targeted interventions in immune cell function represents another promising area of research. Strategies aimed at altering macrophage polarization, reducing M1-mediated inflammation, and promoting anti-inflammatory M2 phenotypes have shown potential in preclinical studies. These approaches seek to disrupt the chronic inflammatory cycle associated with obesity and restore metabolic homeostasis [6,18].

Furthermore, precision medicine has emerged as a key perspective in the management of obesity, enabling the identification of specific metabolic and inflammatory profiles to guide individualized therapeutic interventions. Advances in molecular biology and omics technologies have facilitated the characterization of distinct phenotypes within the obese population, supporting more targeted and effective treatment strategies [24].

Although precision medicine approaches represent a promising perspective for obesity management, important translational challenges remain. The identification of individualized inflammatory and metabolic signatures still depends on complex molecular analyses, high-cost technologies, and limited clinical accessibility, which may restrict their applicability in routine healthcare settings. Furthermore, the heterogeneity of obesity phenotypes and the dynamic interaction between metabolic, hormonal, and immunological pathways make it difficult to establish universally applicable therapeutic models. Therefore, further longitudinal and translational investigations are necessary to validate precision-based strategies and improve their integration into clinical practice [17,24,29].

Another rapidly expanding field involves the development of anti-inflammatory therapies specifically targeting obesity-associated immunometabolic dysfunction. Recent investigations have explored the therapeutic potential of cytokine modulation, inflammasome inhibition, and immune signaling regulation as strategies capable of attenuating chronic low-grade inflammation and improving metabolic homeostasis [21]. Although many of these approaches remain under investigation, they represent promising perspectives for the future management of obesity-related cardiometabolic disease.

Particular attention has also been directed toward the role of incretin-based therapies beyond glycemic control. In addition to their well-established metabolic effects, GLP-1 receptor agonists appear to exert direct vascular and anti-inflammatory actions, including reductions in oxidative stress, endothelial activation, and pro-inflammatory cytokine production [30]. These findings reinforce the growing concept that obesity therapies should not be viewed exclusively through the lens of weight reduction, but rather as comprehensive immunometabolic interventions.

Similarly, combined therapeutic approaches integrating pharmacological treatment, lifestyle modification, and behavioral interventions have demonstrated superior long-term efficacy compared with isolated interventions. Multidisciplinary obesity management programs may improve treatment adherence, reduce inflammatory burden, and enhance cardiometabolic outcomes, particularly in patients with severe visceral obesity and metabolic dysfunction [31].

The incorporation of artificial intelligence and predictive metabolic modeling into obesity management has also emerged as a future direction in precision medicine. Machine learning algorithms and omics-based analyses may contribute to the identification of individualized inflammatory and metabolic signatures, enabling earlier risk prediction and more targeted therapeutic decision-making [29]. These technological advances may substantially transform the future landscape of obesity prevention and treatment.

**Figure 3:**
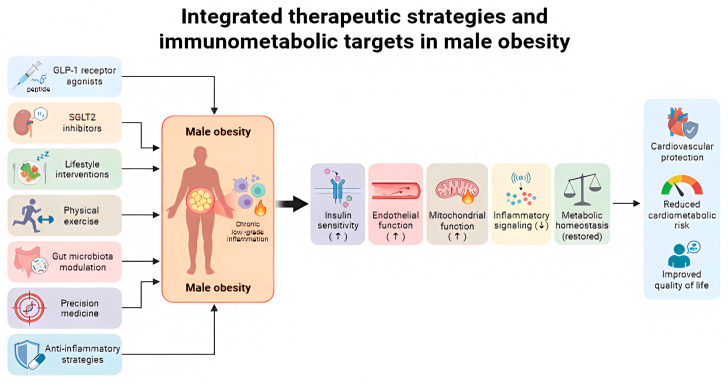
Integrated therapeutic strategies and immunometabolic targets in male obesity. Pharmacological and non-pharmacological interventions, including GLP-1 receptor agonists, SGLT2 inhibitors, lifestyle interventions, physical exercise, gut microbiota modulation, precision medicine, and anti-inflammatory strategies, may improve insulin sensitivity, endothelial function, mitochondrial function, inflammatory signaling, and metabolic homeostasis, contributing to cardiovascular protection and reduced cardiometabolic risk. Created in BioRender. Medeiros, R. (2026) https://app.biorender.com/illustrations/69fd55c16b1c31fc3998061d (accessed on 29 May 2026).

The integrated relationship between emerging therapeutic strategies and immunometabolic targets in male obesity is illustrated in Figure 3, highlighting the potential translational impact of combined metabolic, anti-inflammatory, and precision-based interventions.

The interplay between gut microbiota, inflammation, and metabolism is also gaining increasing attention as an emerging field. Alterations in gut microbial composition have been associated with increased intestinal permeability, systemic inflammation, and metabolic dysregulation. Therapeutic strategies targeting the microbiota, including dietary interventions, probiotics, and prebiotics, may offer new avenues for modulating the inflammatory and metabolic consequences of obesity [24,32,33].

Overall, these emerging approaches reinforce the concept that the future of obesity management lies in integrated strategies that address both metabolic and immunological dimensions, paving the way for more effective and personalized interventions.

The main emerging therapeutic strategies and their potential immunometabolic targets are summarized in Table 3.

## 6. Conclusions

Male obesity represents a complex condition characterized by the dynamic interplay of inflammatory, metabolic, and hormonal mechanisms, all of which significantly contribute to increased cardiometabolic risk. A comprehensive understanding of these mechanisms supports a more integrated and targeted clinical approach, moving beyond interpretations restricted solely to excess body weight.

The incorporation of inflammatory biomarkers and hormonal assessment, combined with pathophysiology-based therapeutic strategies, may represent an important advancement in the management of this condition. Future studies should further elucidate the underlying molecular pathways and foster the development of innovative therapeutic approaches, particularly those involving incretin-based therapies, immunometabolic modulation, and strategies guided by more precise biological profiling.

Despite the comprehensive scope of this review, some limitations should be acknowledged. As a narrative review, the study does not follow the strict methodological rigor of a formal systematic review or meta-analysis, which may increase the risk of selection bias. In addition, the included studies presented considerable heterogeneity regarding study design, population characteristics, inflammatory biomarkers, and clinical outcomes, which may limit direct comparisons between findings. Variability in methodological approaches and differences in obesity phenotyping also represent important challenges for the interpretation and generalization of the available evidence. Nevertheless, this review provides an updated and integrative perspective on the complex immunometabolic mechanisms associated with male obesity and cardiometabolic risk.

Furthermore, recognizing male obesity as a condition strongly influenced by immunometabolic interactions reinforces the need for a paradigm shift in both research and clinical practice. Future investigations should prioritize longitudinal and translational studies capable of elucidating causal pathways and identifying novel biomarkers that may improve early diagnosis and therapeutic monitoring. In addition, integrating metabolic, hormonal, and inflammatory parameters into clinical decision-making may contribute to more precise and individualized management strategies, ultimately improving patient outcomes and reducing the burden of CMDs.

## Figures and Tables

**Table 1:** Inflammatory and Immunometabolic Mechanisms Linking Male Obesity to Cardiometabolic Risk.

Pathophysiological Axis	Main Mechanisms	Principal Molecular Mediators	Metabolic and Vascular Effects	Clinical Consequences
Visceral adiposity	Expansion of metabolically active visceral fat depots	Free fatty acids (FFAs), leptin, adipokines	Lipotoxicity, systemic inflammation, ectopic fat accumulation	Increased cardiometabolic and cardiovascular risk [3,11,12]
Chronic low-grade inflammation	Persistent activation of innate immune pathways	TNF-α, IL-6, CRP, IL-1β	Sustained inflammatory signaling and metabolic dysregulation	Atherosclerosis and metabolic syndrome progression [6,11,21]
NLRP3 inflammasome activation	Activation of intracellular inflammatory complexes	IL-1β, IL-18	Amplification of IR and endothelial dysfunction	Higher risk of diabetes and vascular injury [13,14]
Insulin resistance	Impaired insulin receptor signaling	NF-κB, JNK pathways	Reduced glucose uptake and compensatory hyperinsulinemia	Type 2 diabetes mellitus and metabolic syndrome [7,19]
Endothelial dysfunction	Reduced nitric oxide bioavailability and oxidative injury	ROS, inflammatory cytokines	Vascular stiffness and impaired vasodilation	Hypertension and atherosclerotic disease [3,19]
Adipokine imbalance	Dysregulated secretion of adipose-derived mediators	Reduced adiponectin, increased leptin	Loss of anti-inflammatory protection and vascular impairment	Enhanced cardiometabolic dysfunction [7,12]
Hormonal dysregulation	Obesity-associated functional hypogonadism	Reduced testosterone	Increased visceral adiposity and inflammatory burden	Worsening metabolic and cardiovascular profile [4,5,20]
Immune cell infiltration	Recruitment of inflammatory immune cells	M1 macrophages, neutrophils, T lymphocytes	Sustained adipose tissue inflammation	Chronic metabolic and vascular injury [15,18]
Oxidative stress	Excessive production of reactive oxygen species	ROS, mitochondrial dysfunction	Cellular damage and inflammatory amplification	Cardiometabolic complications [16]
Ectopic fat deposition	Lipid accumulation in non-adipose organs	Lipotoxic intermediates	Organ-specific metabolic dysfunction	NAFLD, cardiac remodeling, IR [17,22,23]

Source: Developed by the authors based on the reviewed literature. Abbreviations: FFAs, free fatty acids; CRP, C-reactive protein; IR, insulin resistance; ROS, reactive oxygen species; NAFLD, nonalcoholic fatty liver disease; TNF-α, tumor necrosis factor alpha; IL, interleukin; NF-κB, nuclear factor kappa B; JNK, c-Jun N-terminal kinase.

**Table 2:** Clinical Biomarkers and Cardiometabolic Implications Associated with Male Obesity.

Biomarker/Clinical Parameter	Pathophysiological Association	Main Clinical Implications	Potential Clinical Utility
C-reactive protein (CRP)	Marker of systemic low-grade inflammation	Increased cardiovascular risk and endothelial dysfunction	Cardiometabolic risk stratification [2,6]
Interleukin-6 (IL-6)	Chronic inflammatory activation and IR	Progression of metabolic syndrome and vascular inflammation	Monitoring inflammatory burden [6,11,21]
Tumor necrosis factor-alpha (TNF-α)	Activation of inflammatory signaling pathways	Impaired insulin signaling and endothelial injury	Assessment of inflammatory activity [7,21]
Reduced adiponectin levels	Loss of anti-inflammatory and insulin-sensitizing effects	Increased IR and atherosclerotic risk	Evaluation of metabolic dysfunction [7,12]
Elevated leptin levels	Sympathetic activation and pro-inflammatory signaling	Hypertension and vascular dysfunction	Identification of obesity-related inflammation [3,12]
Testosterone deficiency	Obesity-associated hypogonadism	Increased visceral adiposity and metabolic deterioration	Hormonal assessment in male obesity [5,20,25]
Waist circumference/Waist-to-hip ratio	Visceral fat accumulation	Higher cardiometabolic vulnerability	Improved obesity phenotype classification [1,2,24]
Insulin resistance markers (HOMA-IR)	Altered glucose metabolism	Type 2 diabetes mellitus and metabolic syndrome	Early metabolic risk identification [7,17]
Oxidative stress markers	Excess ROS production	Endothelial dysfunction and tissue damage	Evaluation of cardiometabolic progression [16]
Endothelial dysfunction indicators	Reduced nitric oxide bioavailability	Atherosclerosis and hypertension	Cardiovascular risk prediction [3,19]

Source: Developed by the authors based on the reviewed literature. Abbreviations: CRP, C-reactive protein; IL, interleukin; IR, insulin resistance; TNF-α, tumor necrosis factor alpha; HOMA-IR, Homeostatic Model Assessment for Insulin Resistance; ROS, reactive oxygen species.

**Table 3:** Emerging Therapeutic Strategies and Immunometabolic Targets in Male Obesity.

Therapeutic Strategy	Main Mechanism of Action	Immunometabolic Effects	Potential Clinical Benefits
GLP-1 receptor agonists	Appetite regulation and delayed gastric emptying	Reduction in systemic inflammation and improvement of insulin sensitivity	Weight reduction and cardiovascular protection [27,28,30]
SGLT2 inhibitors	Increased urinary glucose excretion	Improvement of metabolic homeostasis and reduction of oxidative stress	Cardiovascular and renal protection [28,30]
Lifestyle interventions	Caloric restriction and increased energy expenditure	Decreased inflammatory cytokine production	Improvement of metabolic profile and endothelial function [2,31]
Testosterone replacement therapy	Restoration of androgen deficiency	Reduction in visceral adiposity and inflammatory burden	Improved body composition and metabolic control [20,25]
Gut microbiota modulation	Restoration of intestinal microbial balance	Reduced intestinal permeability and systemic inflammation	Potential cardiometabolic risk reduction [32,33]
Anti-inflammatory therapies	Modulation of inflammatory signaling pathways	Inhibition of cytokine-mediated metabolic dysfunction	Reduction in chronic low-grade inflammation [21]
Precision medicine approaches	Individualized metabolic and molecular profiling	Personalized therapeutic targeting	Optimization of long-term treatment outcomes [24,29]
Macrophage polarization modulation	Reduction in M1 inflammatory activation	Restoration of adipose tissue immune homeostasis	Attenuation of obesity-associated inflammation [15,18]

Source: Developed by the authors based on the reviewed literature. Abbreviations: GLP-1, glucagon-like peptide-1; SGLT2, sodium–glucose cotransporter-2.

## Data Availability

No new data were created or analyzed in this study. Data sharing is not applicable to this article.

## Supplementary Materials

The following supporting information can be downloaded at: https://www.mdpi.com/article/10.3390/biomedicines14071414/s1, Table S1. Main Studies Addressing Immunometabolic Mechanisms and Cardiometabolic Risk in Male Obesity.
