# Supplementary material for: Male Obesity and Cardiometabolic Risk: Inflammatory Mechanisms and Clinical Implications
Source: Biomedicines. 2026 Jun 23;14(7):1414. doi: 10.3390/biomedicines14071414 (PMC13403745; doi:10.3390/biomedicines14071414)
Supplement: Supplementary file 1 [file biomedicines-14-01414-s001.zip › biomedicines-4341976-supplementary.pdf]

Supplementary Table S1. Main Studies Addressing Immunometabolic Mechanisms and Cardiometabolic Risk in Male Obesity

Author / Year	Study Type	Main Topic	Principal Findings	Clinical / Mechanistic Relevance
Hotamisligil, 2017 [32]	Narrative review	Immunometabolism and obesity	Chronic low-grade inflammation plays a central role in obesity-related metabolic dysfunction	Established foundational concepts linking inflammation and metabolic disease
Koenen et al., 2021 [3]	Review article	Adipose tissue and vascular dysfunction	Visceral adiposity contributes to endothelial dysfunction and systemic inflammation	Reinforced cardiovascular implications of obesity
Li et al., 2022 [7]	Review article	Insulin resistance mechanisms	NF-κB and JNK pathways contribute to impaired insulin signaling	Clarified molecular pathways involved in metabolic dysfunction
Engin, 2024 [6]	Narrative review	Adipose tissue inflammation	Obesity-associated inflammation involves immune and metabolic interactions	Expanded the immunometabolic understanding of obesity
Swanson et al., 2019 [20]	Review article	NLRP3 inflammasome	Inflammasome activation amplifies chronic inflammatory signaling	Highlighted the role of innate immunity in obesity
Bhatti et al., 2017 [23]	Review article	Oxidative stress and mitochondrial dysfunction	Excess ROS production contributes to metabolic and vascular injury	Demonstrated the interaction between oxidative stress and inflammation
Longo et al., 2019 [33]	Review article	Adipose tissue dysfunction	Dysfunctional adipose tissue promotes systemic metabolic complications	Reinforced adipokine imbalance and metabolic impairment
De Silva et al., 2024 [5]	Clinical review	Male hypogonadism	Reduced testosterone levels are associated with visceral adiposity and inflammation	Strengthened the endocrine-metabolic perspective in male obesity
Neeland et al., 2018 [24]	Scientific statement	Cardiometabolic heterogeneity of obesity	Obesity phenotypes exhibit distinct metabolic and inflammatory profiles	Supported individualized cardiometabolic risk stratification
Blüher, 2020 [25]	Review article	Metabolically healthy obesity	Obesity phenotypes differ regarding inflammatory and metabolic burden	Highlighted heterogeneity within obesity-related cardiometabolic risk
Wilding et al., 2021 [16]	Randomized clinical trial	Semaglutide therapy	GLP-1 receptor agonists promoted significant weight	Supported incretin-based therapies in obesity management

			loss and metabolic improvement	
Lincoff et al., 2023 [17]	Clinical trial	Cardiovascular outcomes with semaglutide	Semaglutide reduced cardiovascular events in obesity	Expanded therapeutic implications beyond glycemic control
Nauck et al., 2021 [29]	Review article	GLP-1 receptor agonists	GLP-1 therapies exert anti-inflammatory and vascular protective effects	Reinforced immunometabolic therapeutic potential
Coral et al., 2025 [14]	Precision medicine study	Obesity subclassification	Molecular profiling improves cardiometabolic risk prediction	Supported personalized approaches in obesity management
Karczewski & Snyder, 2018 [31]	Review article	Integrative omics and precision medicine	Omics technologies may improve individualized therapeutic strategies	Reinforced future perspectives involving precision medicine

Source: Developed by the authors based on the reviewed literature.
